# VDR BsmI polymorphism and obesity in vitamin D deficiency among undergraduate students: a cross-sectional study

**DOI:** 10.1007/s11033-026-12633-w

**Published:** 2026-08-21

**Authors:** Débora Alejandra Vasquez Rubio, Isabelle Castagnara Albuquerque, Lyana Feijoó Berro Pivetta, Lauren Alicia Flores Viera dos Santos, Vanessa Rosa Retamoso, Heloísa Nogueira Pedra de Pellegrini, Fernanda Barbisan, Matias Nunes Frizzo, Ana Paula Pesarico, Jacqueline da Costa Escobar Piccoli

**Affiliations:** 1 Graduate Program in Biochemistry, Federal University of Pampa (UNIPAMPA), Uruguaiana, 97508-000 RS Brazil; 2 Research Group in Genetics and Genomics, Federal University of Pampa (UNIPAMPA), Uruguaiana, 97501-970 RS Brazil; 3 Undergraduate student, Pharmacy, Federal University of Pampa (UNIPAMPA), Uruguaiana, 97501-970 RS Brazil; 4 Graduate Program of Pharmacology, Federal University of Santa Maria (UFSM), RS 97105-900 Santa Maria, Brazil; 5 Graduate Program of Gerontology, Federal University of Santa Maria (UFSM), RS 97105-900 Santa Maria, Brazil; 6 Graduate Program in Comprehensive Health Care, Regional University of Northwestern Rio Grande do Sul (UNIJUÍ), Ijuí, 98700-000 RS Brazil

**Keywords:** Vitamin D, Obesity, BsmI polymorphism, Vitamin D receptor, Skin color/race, Undergraduate students

## Abstract

**Background:**

Undergraduate students constitute a relevant population for investigating genetic and body weight-related factors associated with vitamin D deficiency. This study aimed to assess the associations of the VDR BsmI single nucleotide polymorphism (SNP) and body weight status, including obesity, with vitamin D deficiency in undergraduate students.

**Methods and Results:**

This cross-sectional study included biochemical and genetic assessment conducted between April and May 2023. A total of 189 students were evaluated after excluding individuals who had received vitamin D supplementation within the six months preceding data collection. SNP genotyping was performed by qPCR using TaqMan probes, vitamin D levels were measured by chemiluminescent immunoassay, and body weight classification was determined according to World Health Organization (WHO) criteria. Vitamin D deficiency was observed in 34.9% of the participants. Individuals with vitamin D deficiency had significantly higher body weight, waist and hip circumference, and body fat percentage than those with sufficient vitamin D levels. The distributions of the BsmI genotypes and alleles differed significantly between the vitamin D groups. Multivariate logistic regression showed that obesity (OR = 4.260; 95% CI: 1.656–10.959) and the recessive genetic model (OR = 4.361; 95% CI: 1.200–15.846) were independently associated with vitamin D deficiency.

**Conclusions:**

Obesity and the VDR BsmI recessive genetic model were independently associated with higher odds of vitamin D deficiency in undergraduate students. These findings highlight the concurrent relevance of genetic and body weight-related factors to vitamin D status and support further research to inform preventive strategies in this population.

## Introduction

Vitamin D deficiency is a global public health concern and has been associated with a wide range of conditions, including autoimmune, osteometabolic, neoplastic, infectious, and cardiovascular diseases [1–4]. In addition to its classical role in calcium and bone metabolism, vitamin D is involved in inflammatory regulation and glucose–insulin homeostasis, which are key processes in cardiometabolic health [5–7].

**Fig. 1:**
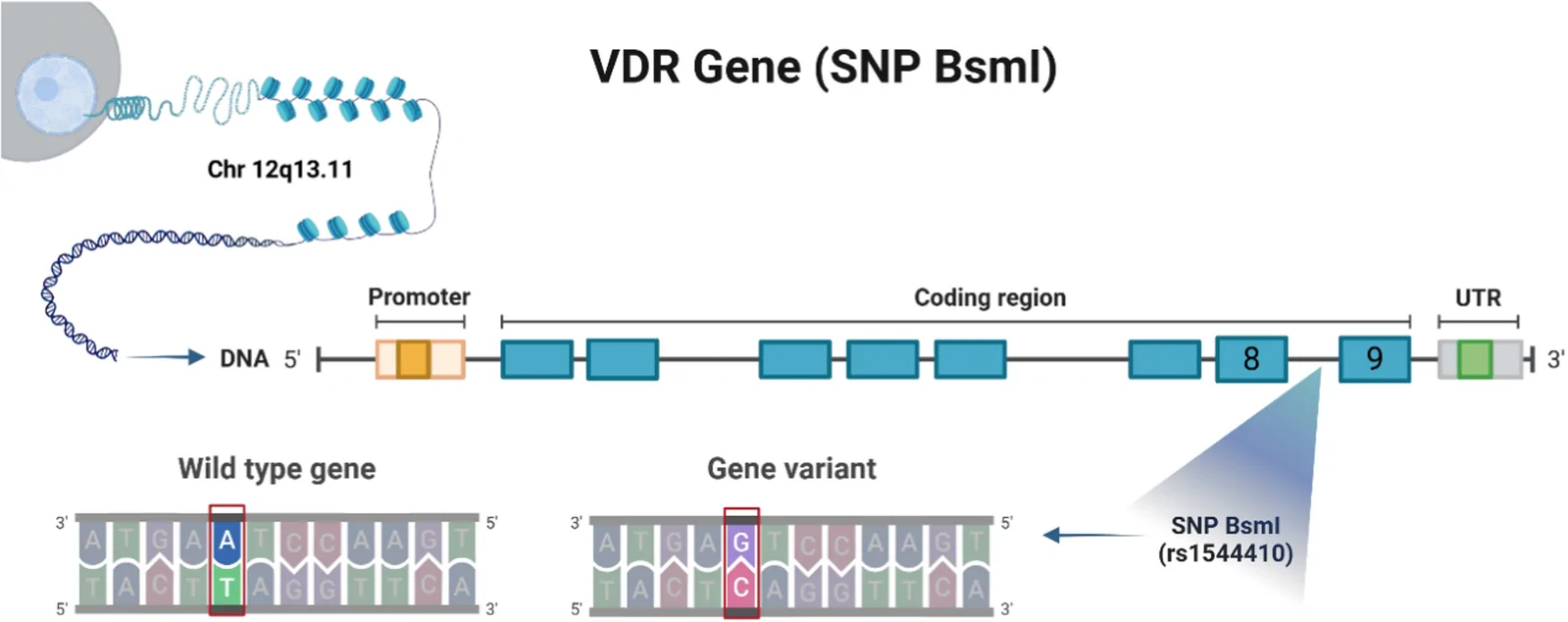
BsmI polymorphism of the vitamin D receptor gene

The biological effects of vitamin D are mediated by the vitamin D receptor (VDR), a nuclear transcription factor that regulates the expression of several target genes [8]. Genetic variations in the VDR gene, particularly single nucleotide polymorphisms (SNPs), may contribute to interindividual variability in vitamin D-related outcomes [5, 9]. Among these variants, the BsmI polymorphism (B > b) has been widely investigated in relation to vitamin D status and metabolic outcomes (Fig. 1) [5, 9, 10]. For the BsmI polymorphism, the B and b alleles correspond to the A and G alleles, respectively, resulting in the BB (A/A), Bb (A/G), and bb (G/G) genotypes.

Obesity is another important factor associated with vitamin D deficiency. Individuals with obesity tend to have lower circulating 25(OH)D concentrations, potentially due to volumetric dilution, sequestration in adipose tissue, reduced bioavailability and differences in sun exposure [3, 11–13]. Additionally, obesity is characterized by chronic low-grade inflammation and adipose tissue dysfunction, which may affect vitamin D metabolism and VDR-mediated signaling pathways [14, 15].

VDR polymorphisms have also been investigated in relation to vitamin D status, obesity, and metabolic phenotypes. However, findings remain heterogeneous, and evidence in young populations is still limited [5, 16, 17]. Undergraduate students represent a relevant population for investigation because dietary habits, physical inactivity, and limited sun exposure may contribute to inadequate vitamin D status [18–20]. Therefore, this study aimed to evaluate the associations of the VDR BsmI polymorphism and obesity with vitamin D deficiency among undergraduate students.

## Materials and methods

### Study population

This study adopted an analytical cross-sectional epidemiological design. Ethical approval was obtained from the UNIPAMPA Research Ethics Committee, under reference number 5.854.845. Participants were undergraduate students from the Federal University of Pampa (UNIPAMPA), Uruguaiana campus, located in Uruguaiana, Rio Grande do Sul, southern Brazil (29°45′S). Data collection and participant sampling occurred between April and May 2023, corresponding to autumn in the Southern Hemisphere.

An a priori sample size calculation was performed based on an estimated campus population of 2,460 students, considering a 95% confidence level and a 5% margin of error. The estimated sample size was 333 participants. Undergraduate students aged 18 years or older were invited to participate through social media, posters displayed on campus, and oral invitations made in classrooms. Before enrollment, participants were informed about the study procedures, risks, and benefits and provided written informed consent. A total of 244 undergraduate students were initially recruited. Vitamin D supplementation during the six months preceding data collection was the sole exclusion criterion. After applying this criterion, the final analytical sample comprised 189 participants. Biological sex was self-reported as female or male.

### Sociodemographic and lifestyle data

A structured questionnaire was administered to collect information on sociodemographic characteristics and lifestyle factors of the respondents. Additionally, skin color/race was determined by self-declaration and classified according to the Brazilian Institute of Geography and Statistics (IBGE) [21]. Individuals who self-declared as black or brown were grouped into the black category.

### Anthropometric and hemodynamic assessments

Using a digital bioimpedance scale for whole body HBF-514 Omron Healthcare Co (Kyoto, Japan), the following were measured: weight, Body Mass Index (BMI), percentage of lean mass, body fat percentage, and visceral fat. Height, waist circumference, and hip circumference were measured with a tape measure. Systolic blood pressure (SBP) and diastolic blood pressure (DBP) were determined using a standard digital sphygmomanometer after a 15-minute rest period, with participants in a seated position. A single blood pressure measurement was obtained for each participant and used in the analyses.

### Body weight classification

Body weight classification followed the World Health Organization (WHO) criteria [22], which uses BMI to define body weight categories. Accordingly, underweight was defined as BMI < 18.5 kg/m², normal weight as BMI 18.5–24.9 kg/m², overweight as BMI 25–29.9 kg/m², and obesity as BMI ≥ 30 kg/m².

### Biochemical assessments

Participants underwent venous blood collection after a 12-hour fast. Samples were centrifuged for 15 min at 3000 rpm, and aliquots of plasma and serum were stored at -20 °C for later analyses. Serum concentrations of cholesterol, glucose, triglycerides, high-density lipoprotein (HDL), calcium, Alanine Aminotransferase (ALT), and Aspartate Aminotransferase (AST) were measured using a semi-automated ChemWell BioRad^®^ instrument with the commercial Labtest^®^ kit (Lagoa Santa, MG, Brazil). Serum insulin concentration was analyzed by chemiluminescence using the Bioclin^®^ (Belo Horizonte, MG, Brazil) commercial kit.

Low-density lipoprotein (LDL) concentrations were calculated according to the Friedewald equation [23]. Serum vitamin D was determined by a chemiluminescent immunoassay. Insulin resistance was estimated using the Homeostatic Model Assessment for Insulin Resistance (HOMA-IR), calculated as fasting insulin (µU/mL) × fasting glucose (mg/dL) / 405. In accordance with the reference ranges for vitamin D established by the Brazilian Society of Endocrinology and Metabolism (SBEM), a cutoff of 20 ng/mL was established for serum vitamin D levels [24]. Accordingly, the total sample was divided into two groups: vitamin D < 20 ng/mL (VitD < 20) and vitamin D ≥ 20 ng/mL (VitD ≥ 20).

### DNA extraction and genotyping

Genomic DNA was extracted from total venous blood leukocytes using the PureLink™ Genomic DNA Kit (Invitrogen™, Carlsbad, CA, USA). The obtained material was stored at -20 °C until analysis. To verify the presence of genomic DNA, 1% agarose gel electrophoresis in TRIS-borate-EDTA (TBE) buffer was performed and documented photographically using a Gel Doc XR + Gel Documentation System (Bio-Rad Laboratories, Inc.).

The VDR gene BsmI polymorphism was identified by real-time polymerase chain reaction (qPCR) with allele discrimination using a predesigned TaqMan SNP Genotyping Assay (ID C___8716062_20; Applied Biosystems, Foster City, CA, USA) on a StepOne™ Real-Time PCR System (Thermo Fisher Scientific, USA). Amplification reactions were performed in a final volume of 10 µL using TaqMan Genotyping Master Mix according to the manufacturer’s instructions. Thermal cycling conditions consisted of an initial denaturation step at 95 °C for 10 min, followed by 40 cycles of 95 °C for 15 s and 60 °C for 1 min. Fluorescence detection was performed at 60 °C, and genotype assignment was analyzed using Data Connect software (Thermo Fisher Scientific, USA). Negative controls and control samples representing all possible genotypes were included in each run. Primer and probe sequences were not available because a proprietary commercial assay was used.

### Statistical analysis

Statistical analyses were performed using the Statistical Package for the Social Sciences (SPSS) version 20.0, IBM Corp (Armonk, NY, USA). Quantitative variables were analyzed using Student’s t-test or one-way or multivariate analysis of variance as appropriate, followed by a Bonferroni post hoc test. Categorical variables were analyzed using the chi-square test. Hardy-Weinberg equilibrium was assessed in the total population using Pearson’s chi-square test with two degrees of freedom, comparing observed and expected genotype frequencies. Normality of data distribution was assessed using the Shapiro–Wilk test.

To evaluate the potential influence of intervening variables on the data (sex, age, and obesity), logistic regression using the Backward Wald method was employed. In the model, all variables with a significance level of *p* < 0.20 in univariate analyses were included. Comparisons with *p* < 0.05 were considered statistically significant.

## Results

### General characteristics of the population

The present study included 189 individuals, with 152 females (80.4%) and 37 males (19.6%), with a mean age of 23.29 ± 4.98 years. Of these, 136 (72.0%) self-identified as white and 53 (28.0%) as black. 65% (*n* = 123) of the students presented VitD ≥ 20 and 35% (*n* = 66) presented VitD < 20. Anthropometric, hemodynamic, and biochemical variables of the studied group are presented in Table 1.

**Table 1:** Age-related, anthropometric, hemodynamic, and biochemical variables in the total group and in the groups classified by the vitamin D cutoff point

Variables	TOTAL (*n* = 189)	VitD < 20 (*n* = 66)	VitD ≥ 20 (*n* = 123)	*p*
**Anthropometric Variables**	Mean ± SD	Mean ± SD	Mean ± SD	
Age (years)	23.29 ± 4.98	24.25 ± 5.40	22.79 ± 4.68	0.056
Weight (kg)	67.05 ± 14.51	71.28 ± 17.22	64.76 ± 12.28	**0.003**
BMI (kg/m^2^)	24.83 ± 5.04	26.54 ± 6.19	23.91 ± 4.04	**0.001**
Waist circumference (cm)	79.29 ± 11.63	83.73 ± 13.47	76.95 ± 9.80	**< 0.001**
Hip circumference (cm)	92.20 ± 10.95	96.09 ± 13.42	90.14 ± 8.77	**< 0.001**
Body fat (%)	34.61 ± 10.17	36.97 ± 9.87	33.36 ± 10.13	**0.020**
**Hemodynamic Variables**				
Systolic Blood Pressure (mmHg)	114.60 ± 20.18	117.03 ± 21.65	113.31 ± 19.31	0.231
Diastolic Blood Pressure (mmHg)	76.22 ± 18.02	79.02 ± 15.51	74.73 ± 19.11	0.122
**Biochemical Variables**				
Vitamin D (ng/mL)	23.40 ± 7.22	15.63 ± 3.28	27.57 ± 4.95	**< 0.001**
Calcium (mg/dL)	8.91 ± 2.15	8.81 ± 1.75	8.97 ± 2.35	0.636
Insulin (µUI/mL)	9.82 ± 5.81	10.79 ± 6.42	9.29 ± 5.40	0.093
Glucose (mg/dL)	95.60 ± 14.66	94.78 ± 11.82	96.04 ± 16.00	0.575
HOMA-IR	2.39 ± 1.65	2.78 ± 1.80	2.22 ± 1.57	**0.034**
Cholesterol (mg/dL)	176.57 ± 34.98	180.66 ± 36.53	174.36 ± 34.05	0.239
Triglycerides (mg/dL)	142.85 ± 56.87	160.87 ± 61.44	133.10 ± 51.94	**0.001**
HDL (mg/dL)	60.00 ± 15.34	60.90 ± 16.27	59.51 ± 14.87	0.544
LDL (mg/dL)	89.42 ± 28.55	89.95 ± 30.79	89.13 ± 27.39	0.851
AST (U/L)	27.43 ± 12.19	28.87 ± 13.20	26.67 ± 11.62	0.281
ALT (U/L)	16.62 ± 8.08	19.36 ± 9.69	15.13 ± 6.65	**0.001**

VitD < 20: group with vitamin D deficiency. VitD ≥ 20: group with sufficient vitamin D. BMI: Body Mass Index. HOMA-IR: Homeostatic Model Assessment for Insulin Resistance. HDL: High-Density Lipoprotein. LDL: Low-Density Lipoprotein. AST: Aspartate Aminotransferase. ALT: Alanine Aminotransferase. The data are presented as mean ± standard deviation (SD). The p value shows differences between the groups VitD < 20 and VitD ≥ 20 as obtained by Student’s t-test. Significant p-values are presented in bold

Table 1 results indicate significant differences in all anthropometric variables between groups classified by the vitamin D cutoff point. Comparisons between groups showed statistically significant differences in body weight (*p* = 0.003), BMI (*p* = 0.001), waist circumference (*p* < 0.001), hip circumference (*p* < 0.001), and body fat (*p* = 0.020).

In the biochemical analyses, between the VitD < 20 and VitD ≥ 20 groups, serum triglyceride levels were higher in the VitD < 20 group (*p* = 0.001), as was ALT (*p* = 0.001). As expected, serum vitamin D levels were significantly lower in the VitD < 20 group (15.63 ± 3.28 ng/mL) than in the VitD ≥ 20 group (27.57 ± 4.95 ng/mL), *p* < 0.001. There were no differences between groups in the other biochemical markers studied. Additionally, participants with VitD < 20 had significantly higher HOMA-IR values than those with sufficient vitamin D levels (2.78 ± 1.80 vs. 2.22 ± 1.57, respectively; *p* = 0.034).

There was no association between participants’ sex and vitamin D cutoff. The chi-square test showed *p* = 0.424, with the sex distribution being similar between groups (VitD < 20: 33.8% female; 66.2% male; VitD ≥ 20: 49.5% female; 50.5% male), corroborating the absence of a significant association between sex and the vitamin D cutoff in the sample. Furthermore, there was no evidence of association between self-reported race/color (Black vs. White) and the vitamin D cutoff, with observed counts indicating a relatively similar distribution between groups (*p* = 0.062).

### Distribution of the BsmI SNP of the VDR gene

Analysis of the BsmI SNP of the VDR gene yielded three genotype variants: BB (A/A), Bb (A/G), and bb (G/G). The homozygous BB was considered the reference genotype according to the literature, as it predisposes to normal vitamin D levels [9]. The sample was categorized using the recessive genetic model, which groups the risk alleles (Bb and bb) against the protective allele (BB).

Table 2 shows the allele and genotype frequencies of the BsmI SNP of the VDR gene, in the total group and, additionally, the allele and genotype frequencies in groups classified by the vitamin D cutoff. The same table also presents the frequencies of the recessive model in the studied groups as well as in the total group. Furthermore, the table includes the Hardy-Weinberg equilibrium test.

**Table 2:** Allele and genotype frequencies and recessive model of the BsmI SNP of the VDR gene in vitamin D groups classified by the cutoff, and results of the chi-square test for Hardy-Weinberg equilibrium

	Total (*n* = 189)	HWE X^2^	*p* value	VitD < 20 (*n* = 66)	VitD ≥ 20 (*n* = 123)	*p* value
	*n* (%)			*n* (%)	*n* (%)	
**Genotypes**						
BB	24 (12.7)			3 (4.5)	21 (17.1)	
Bb	79 (41.8)	0.776	0.679	32 (48.5)	47 (38.2)	0.040*
bb	86 (45.5)			31 (47.0)	55 (44.7)	
**Alleles**						
B	127 (33.6)			38 (28.8)	89 (36.2)	
b	251 (66.4)			94 (71.2)	157 (63.8)	
**Recessive model**						
Bb + bb	165 (87.3)			63 (95.5)	102 (82.9)	0.012*
BB	24 (12.7)			3 (4.5)	21 (17.1)	

HWE: Hardy-Weinberg Equilibrium. VitD < 20: group with vitamin D deficiency. VitD ≥ 20: group with sufficient vitamin D. Data are presented as absolute and relative frequencies. Hardy-Weinberg equilibrium was tested using the χ² test. *Differences between genotype frequencies and the recessive model were tested using Fisher’s exact test. Significant p-values are shown in bold

The genotype frequencies for the BsmI SNP of the VDR gene were consistent with Hardy-Weinberg equilibrium in the total studied group (*p* = 0.679). As observed in Table 2, there was a statistically significant difference in both allele (*p* = 0.012) and genotype (*p* = 0.040) distributions between the groups classified by the vitamin D cutoff. The dominant model was also evaluated; however, no significant associations with vitamin D deficiency were observed (data not shown).

### Vitamin D

**Fig. 2:**
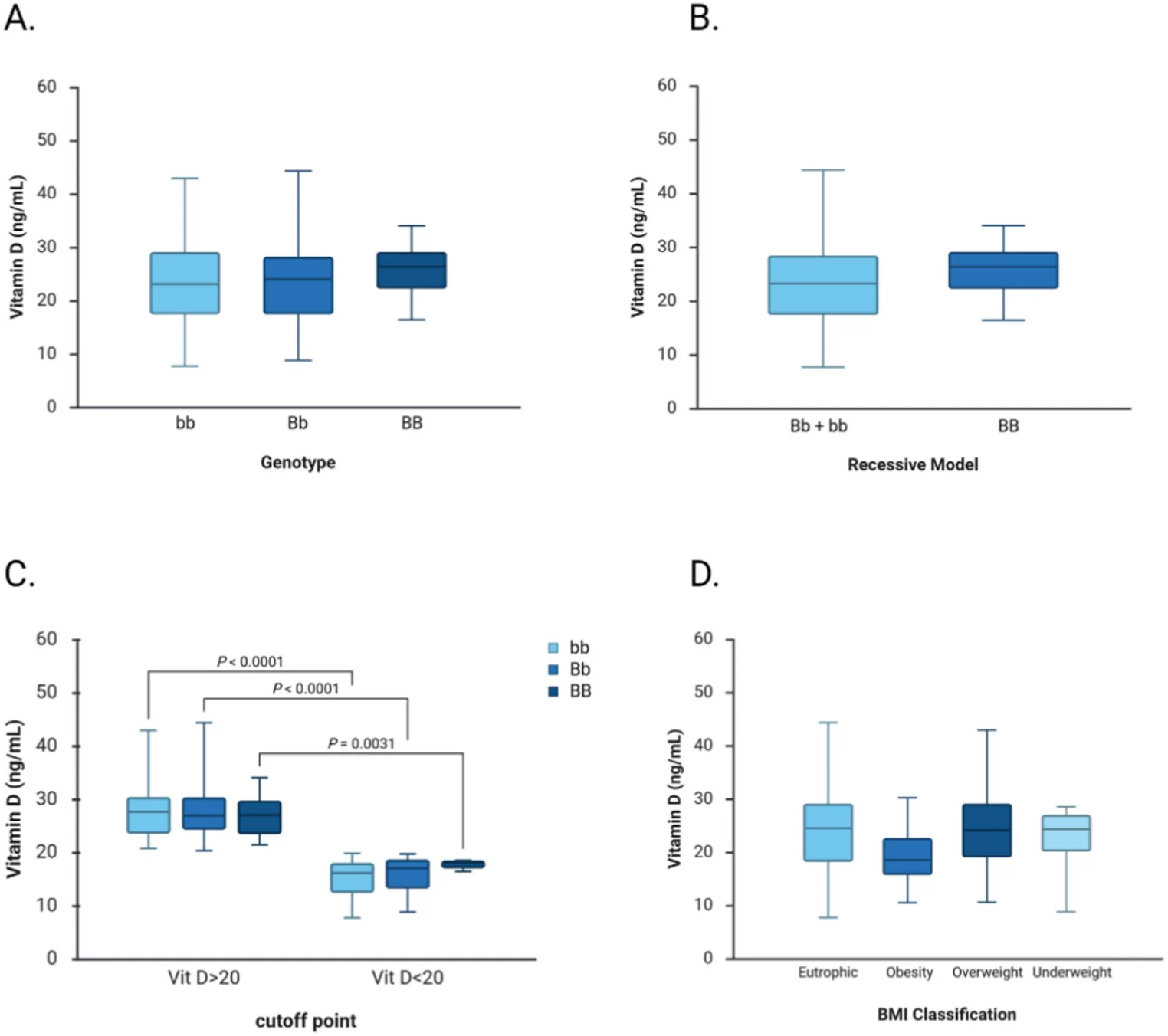
Mean vitamin D levels by BsmI SNP genotypes of the VDR gene (**A**), recessive model (**B**), vitamin D cutoff and genotypes (**C**), and BMI-based classification (**D**). Data are presented as mean ± standard deviation (SD), and error bars represent SD. Differences in mean serum vitamin D levels among BsmI SNP genotypes (**A**) were evaluated using one-way ANOVA. Differences between groups defined according to the recessive genetic model (**B**) were evaluated using an independent-samples t-test. Differences in serum vitamin D levels according to genotype and vitamin D status (**C**) were evaluated using two-way ANOVA followed by Bonferroni-adjusted multiple comparisons. Differences among BMI categories (**D**) were evaluated using one-way ANOVA. Statistical significance was set at *p* < 0.05. Statistically significant pairwise comparisons and their exact p values are shown in panel C; no statistically significant differences were observed in panels A, B, or D

Serum vitamin D levels were analyzed across the different groups studied, and their mean values are presented in Fig. 2. No differences were observed in vitamin D means for the sample stratified by genotypes (Fig. 2a) (BB: 25.7 ± 4.68 ng/mL, Bb: 22.7 ± 7.28 ng/mL, bb: 23.3 ± 7.67, *p* = 0.217), by the recessive model (Fig. 2b) (Bb + bb: 23.07 ± 7.47 ng/mL, BB: 25.70 ± 4.68 ng/mL, *p* = 0.096) or by BMI classification (Fig. 2d) (Underweight: 22.76 ± 6.17 ng/mL, Normal weight: 24.05 ± 7.78 ng/mL, Overweight: 23.82 ± 6.51 ng/mL, Obesity: 19.73 ± 5.54 ng/mL, *p* = 0.070).

When categorized by the cutoff point (Fig. 2c), the statistical analyses indicated significant differences between groups, with *p* < 0.001 for the bb and Bb genotypes and *p* = 0.003 for the BB genotype when compared with each other within the vitamin D cutoff categorization.

### Body weight classification

BMI was used as an obesity indicator in the sample analyzed, and the groups stratified by BMI were analyzed using the vitamin D cutoff. Table 3 presents the frequencies of individuals classified by BMI and their distribution at the vitamin D cutoff.

**Table 3:** Frequency of individuals classified by BMI and distribution at the vitamin D cutoff

BMI Classification	Total (*n* = 189)	VitD < 20 (*n* = 66)	VitD ≥ 20 (*n* = 123)
	*n* (%)	*n* (%)	*n* (%)
Underweight	9 (4.8)	2 (1.1)	7 (3.7)
Normal weight	105 (55.6)	33 (17.5)	72 (38.1)
Overweight	52 (27.5)	16 (8.5)	36 (19.0)
Obesity	23 (12.1)	15 (7.9)	8 (4.2)

BMI: Body Mass Index. VitD < 20: group with vitamin D deficiency. VitD ≥ 20: group with sufficient vitamin D. Data are presented as absolute and relative frequencies

Serum vitamin D means were analyzed in the BMI-classified groups stratified by genotype and by the BsmI SNP recessive model. No statistically significant effect of genotypes (*p* = 0.998) or BMI classification (*p* = 0.796) on serum vitamin D means was observed. Additionally, there was no significant interaction between genotypes and BMI classification on serum vitamin D means (*p* = 0.094). Furthermore, no statistically significant associations were found between the recessive model, BMI classification, and serum vitamin D means (*p* = 0.759).

### Logistic regression analysis

To test the independence of the variables, a multivariate logistic regression analysis was performed. The following variables were included in the model for the outcome of “vitamin D levels < 20 ng/mL”: age, sex, self-reported race/color, obesity, and the recessive model. In the final step, the independent variables were obesity, self-reported race/color, and the recessive model (Table 4).

**Table 4:** Multivariate logistic regression analysis for vitamin D deficiency (< 20 ng/mL)

Variables	OR	95% CI	*p* value
Recessive model	4.361	1.200–15.846	**0.025**
Black race/color	1.884	0.953–3.724	0.069
Obesity	4.260	1.656–10.959	**0.003**

OR: Odds Ratio. 95% CI: 95% confidence interval. Significant p-values are shown in bold

The results indicated that obesity and the recessive genetic model were independently associated with vitamin D deficiency. Self-reported race/color was not statistically associated with vitamin D deficiency in this population.

## Discussion

This study showed that obesity and the VDR BsmI recessive genetic model were independently associated with vitamin D deficiency in undergraduate students. Although no statistically significant interaction between genotype and BMI classification was observed for serum vitamin D levels, the findings support the concurrent contribution of genetic and body weight-related factors to vitamin D status in this population.

Vitamin D plays a central role in inflammatory regulation and glucose–insulin metabolism, both of which are critical components of cardiometabolic risk [25, 26]. In this context, understanding how genetic variation modulates vitamin D status in young adults is essential for early prevention strategies. Although the functional consequences of the BsmI polymorphism remain uncertain, this noncoding variant may be linked to regulatory differences affecting VDR expression or vitamin D-related pathways [9]. At the same time, obesity may reduce circulating 25(OH)D levels due to sequestration in adipose tissue and increased volume of distribution, limiting its bioavailability [11, 13]. These mechanisms may contribute to vitamin D deficiency through distinct biological pathways.

Several mechanisms have been proposed to explain the relationship between obesity and vitamin D deficiency. First, because vitamin D is a fat-soluble molecule, it may become sequestered within adipose tissue, reducing its availability in the circulation [11, 13, 15]. Moreover, individuals with obesity have a larger body volume, which may contribute to a volumetric dilution effect and consequently lower serum 25(OH)D concentrations [11, 12]. Obesity is also characterized by chronic low-grade inflammation, with increased production of pro-inflammatory cytokines and adipokines that may interfere with vitamin D metabolism and VDR-mediated signaling pathways [14, 15, 27]. In addition, adipose tissue dysfunction has been associated with alterations in vitamin D storage, mobilization, and endocrine activity, reinforcing the concept that obesity and vitamin D deficiency are linked through multiple interconnected biological pathways [14, 15, 27]. These pathways provide biological plausibility for the independent associations of obesity and VDR genetic variability with vitamin D deficiency observed in the present study.

The prevalence of vitamin D deficiency observed in this study (34.9%) is consistent with reports of inadequate vitamin D status among university students [20]. Dietary habits, physical inactivity, and limited sun exposure may contribute to the vulnerability of this population [18–20]. This highlights the vulnerability of this group and reinforces the need for targeted preventive measures.

Our findings also indicate a higher frequency of the b allele among participants with vitamin D deficiency, suggesting that this polymorphism may influence susceptibility to inadequate vitamin D status. Moreover, the recessive genetic model remained independently associated with vitamin D deficiency in the multivariate analysis. Similar associations have been described in previous studies investigating VDR gene variability and metabolic outcomes [5, 10].

Previous studies examining VDR polymorphisms in relation to obesity and metabolic phenotypes have reported heterogeneous findings. Chauhan and Medithi observed associations of the BsmI polymorphism with obesity, type 2 diabetes, and other metabolic alterations, supporting a potential role of VDR genetic variability in cardiometabolic susceptibility [17]. In contrast, Karonova et al. found no association between the BsmI polymorphism and anthropometric or biochemical markers of metabolic syndrome in middle-aged women [16]. These differences may reflect variation in age, sex distribution, ethnicity, metabolic profile, and the outcomes investigated across study populations. In the present study, the BsmI recessive genetic model was associated with vitamin D deficiency, whereas the genotype-by-BMI interaction was not statistically significant, suggesting independent rather than interactive contributions of these factors.

Furthermore, vitamin D deficiency has been linked to insulin resistance, dyslipidemia, and increased risk of type 2 diabetes, emphasizing its broader metabolic implications. Participants with vitamin D deficiency also exhibited higher HOMA-IR values, indicating a less favorable insulin resistance profile in this group. This finding is consistent with evidence linking vitamin D deficiency to alterations in glucose–insulin homeostasis [28, 29]. However, given the cross-sectional design, this finding should be interpreted as an association and does not establish a causal relationship between vitamin D status and insulin resistance.

The multivariate analysis showed a strong independent association between obesity and vitamin D deficiency (OR = 4.260; 95% CI 1.656–10.959), supporting the well-established relationship between adiposity and reduced vitamin D availability [12, 27, 30]. As a fat-soluble vitamin, vitamin D is stored in adipose tissue, which may limit its release into circulation in individuals with excess adiposity [13, 15]. Similarly, the recessive genetic model was independently associated with vitamin D deficiency (OR = 4.361; 95% CI 1.200–15.846), suggesting that genetic predisposition may increase susceptibility to vitamin D deficiency. This finding raises the possibility that VDR polymorphisms influence not only vitamin D levels but also the biological response to this hormone, potentially modifying downstream metabolic pathways [17].

The observed association between elevated ALT levels and vitamin D deficiency further supports the link between vitamin D status and metabolic health. Alterations in liver enzymes may reflect underlying metabolic disturbances, including non-alcoholic fatty liver disease, which has been associated with vitamin D deficiency [31, 32].

Despite these findings, some limitations should be considered. The cross-sectional design does not allow causal relationships to be established. In addition, data on dietary vitamin D intake and sun exposure were not available. Data collection was conducted during autumn, which may have influenced absolute serum vitamin D levels and the observed prevalence of vitamin D deficiency. Nevertheless, because all participants were evaluated during the same period, seasonality is less likely to have substantially affected the internal comparisons among genotypes and BMI categories. Although an a priori sample size calculation indicated a target of 333 participants, the initial recruitment included 244 students, and the application of the eligibility criteria resulted in a final analytical sample of 189 participants. Therefore, the smaller analytical sample may have reduced the statistical power to detect smaller associations or interaction effects and may limit the generalizability of the findings. The predominance of female participants may limit the generalizability of the findings, although sex was included as a potential adjustment variable in the multivariate analysis. Additionally, blood pressure was assessed using a single measurement, which may have increased measurement variability. However, blood pressure was evaluated as a descriptive cardiometabolic variable and was not central to the primary associations investigated in this study. Future studies integrating genetic, environmental, and behavioral variables are needed to better understand the multifactorial determinants of vitamin D deficiency.

From a public health perspective, these findings reinforce the relevance of addressing obesity and inadequate vitamin D status among undergraduate students. General health promotion strategies involving physical activity, balanced nutrition, and appropriate sun exposure may support vitamin D and metabolic health [18–20]. Overall, the results highlight the concurrent relevance of genetic variation and obesity to vitamin D status and support further investigation in university populations.

## Conclusion

In this cross-sectional study, obesity and the VDR BsmI recessive genetic model were independently associated with higher odds of vitamin D deficiency in undergraduate students. Participants with vitamin D deficiency also exhibited higher HOMA-IR values, indicating a less favorable insulin resistance profile. Although no statistically significant interaction between genotype and BMI classification was observed, the findings highlight the concurrent relevance of genetic and body weight-related factors to vitamin D status. These results should be interpreted in light of the cross-sectional design and the other study limitations. Larger longitudinal studies involving more diverse populations are needed to confirm these associations and clarify the biological mechanisms underlying vitamin D deficiency.

## Data Availability

No datasets were generated or analysed during the current study.
